# *In-Vivo* Measurement of Muscle Tension: Dynamic Properties of the MC Sensor during Isometric Muscle Contraction

**DOI:** 10.3390/s140917848

**Published:** 2014-09-25

**Authors:** Srđan Đorđević, Sašo Tomažič, Marco Narici, Rado Pišot, Andrej Meglič

**Affiliations:** 1 TMG-BMC Ltd., Splitska 5, Ljubljana 1000, Slovenia; E-Mail: andrej.meglic.info@gmail.com; 2 Institute for Kinesiology Research, Science and Research Centre of the University of Primorska, Garibaldijeva 1, Koper 6000, Slovenia; E-Mail: rado.pisot@zrs.upr.si; 3 Faculty of Electrical Engineering, University of Ljubljana, Tržaška 25, Ljubljana 1000, Slovenia; E-Mail: saso.tomazic@fe.uni-lj.si; 4 University of Nottingham, School of Graduate Entry Medicine and Health, Derby Royal Hospital, Uttoxeter Road, Derby DE22 3DT, UK; E-Mail: marco.narici@nottingham.ac.uk

**Keywords:** muscle force, muscle tension, noninvasive, selective, *in vivo*, measurement

## Abstract

Skeletal muscle is the largest tissue structure in our body and plays an essential role for producing motion through integrated action with bones, tendons, ligaments and joints, for stabilizing body position, for generation of heat through cell respiration and for blood glucose disposal. A key function of skeletal muscle is force generation. Non-invasive and selective measurement of muscle contraction force in the field and in clinical settings has always been challenging. The aim of our work has been to develop a sensor that can overcome these difficulties and therefore enable measurement of muscle force during different contraction conditions. In this study, we tested the mechanical properties of a “Muscle Contraction” (MC) sensor during isometric muscle contraction in different length/tension conditions. The MC sensor is attached so that it indents the skin overlying a muscle group and detects varying degrees of tension during muscular contraction. We compared MC sensor readings over the biceps brachii (BB) muscle to dynamometric measurements of force of elbow flexion, together with recordings of *surface EMG* signal of BB during isometric contractions at 15° and 90° of elbow flexion. Statistical correlation between MC signal and force was very high at 15° (r = 0.976) and 90° (r = 0.966) across the complete time domain. Normalized SD or σ*_N_* = σ/max(***F***_MC_) was used as a measure of linearity of MC signal and elbow flexion force in dynamic conditions. The average was 8.24% for an elbow angle of 90° and 10.01% for an elbow of angle 15°, which indicates high linearity and good dynamic properties of MC sensor signal when compared to elbow flexion force. The next step of testing MC sensor potential will be to measure tension of muscle-tendon complex in conditions when length and tension change simultaneously during human motion.

## 1. Introduction

Skeletal muscle system is the largest human tissue structure. Our muscles are a biological force generator specifically designed to provide the functional requirements for posture and movement. Muscle force is generated by the active (cross bridge) muscle elements and exerted via passive elastic components, such as fascia and tendon, both in series and in parallel. Measuring and understanding the mechanical properties of the muscle-tendon complex in different task conditions is an important and ongoing challenge for scientists and clinicians interested in muscle function in healthy and diseased individuals. Almost all muscle function measurements are in fact joint moment measurements typically resulting from the composite force output of multiple agonist and antagonist muscles, rather than direct muscle force measurements from a target muscle [1]. The earliest approximations of muscle force were based on torque measurements measured using a dynamometer and then estimating the moment arm using radiographs [2], followed by MRIs [3] and finally, in the most recent implementation, dynamic ultrasound imaging to make more precise moment arm estimations during muscle contraction and therefore enabling more accurate muscle force calculations. However, these methods still share the problem of composite force measurements, thus limiting application [4,5]. For example, the recovery of a specific hamstring muscle which had suffered partial strain injury can only be inferred from force measurements of knee flexion, which are due to the composite action of all three hamstring muscles, a counter-force from the quadriceps and possibly small contributions from popliteus and gastrocnemius. Direct measurements of the muscle could yield more specific muscle recovery information.

Precise muscle force measurements have been made by applying a transducer surgically placed around a human tendon during voluntary movement, providing muscle force values directly without the need for moment arm calculation [1]. This method was based on work by Gregor [6] in the cat and was applied to humans by Komi [7,8] who later refined the technique using a fiber optic probe inserted in the Achilles tendon.

The latter two methods are selective but invasive, and only measure force in a specific tendon area with the assumption that force is uniformly distributed across the tendon. These methods measure the longitudinal muscle-tendon complex tension, but the possibilities of developing a greater understanding of dynamic muscle function, using non-invasive and selective sensors measuring perpendicular and axial tension of muscle-tendon complex have not been explored. Two new studies from 2013 [9,10] on myofilament level confirm the importance of radial (perpendicular) displacement in understanding FT relation and energy storage during muscle contraction and relaxation.

An additional aspect of interest is the dynamic characteristics of skeletal muscle force/tension development. The rate of rise in Contractile Force measured under Isometric Conditions (CFIC) has been identified as an important parameter that quantifies the ability of the neuromuscular system to produce so-called “explosive” muscle actions [11–15]. CFIC is influenced by both muscle properties [16,17] and neural factors in the initial phase of contraction [11,13,18,19]. CFIC is also associated with the compliance of the passive force-transmitting structures [20]. Such an association has been proposed in more recent studies [21–24].

Estimates of muscle force based on surface electromyography (EMG) measurements have also been proposed [25]. Muscle force (active elements) is mainly determined by the number of active motor units (the EMG signal is the summation of motor unit potentials), their size (cross-sectional area), and their firing rate [25,26]. The validity of force estimates is limited by a range of factors inherent to the EMG signal and to physiology and anatomy of the muscle [25], and by the fact that determination of tension/force load in muscle-tendon complex cannot be estimated with surface EMG without additional mechanical information about length and passive properties of the muscle-tendon complex. The net estimation has, at best, a low-moderate correlation with force.

A broad application range of the methods to measure isometric muscle contraction tension/force (muscle biomechanics, functional muscle diagnostics, and sports sciences, training optimization, *etc.*) has been an additional reason for developing a new mechanical sensor concept for selective, non-invasive and *in situ* measurement methods. In 2011 [27], a novel non-invasive muscle tension isometric measurement method with muscle contraction (MC) sensor was reported. In the study, a high correlation between elbow peak flexion force at five target levels—10%, 30%, 50%, 70%, and 90% of maximum voluntary contraction (MVC)—and MC sensor measured peak tension was observed for the biceps' brachii (BB) muscle.

In this paper, we further evaluate the MC sensor by examining its properties and behaviour in the time domain. The aim of this study was to investigate the MC sensor dynamic properties, *i.e.*, how the response of the sensor correlated with the force produced by the BB muscle in the time domain during increasing and decreasing isometric contractions of BB. For repeatability of measurement, we used the same length and area of the sensor tip, which have proved successful in static measurements. We performed measurements for two different (nearly extreme) elbow angles: 15° and 90°, with different initial tensions of the observed muscle, and compared signals of the sensor response to the signals obtained from dynamometer and EMG, simultaneously. We used standard deviation of MC signals from normalized force signals as a measure of the sensor linearity. A low standard deviation is a good indicator of linearity.

## 2. Subjects and Methods

Sixteen volunteers (10 males and 6 females), ranging between 20 and 50 years of age (32.6 ± 9.8 years; all values represent the mean ± SD unless indicated otherwise) participated in the study. Subcutaneous fat was measured using a skin fold calliper. All of the subjects were healthy and had no known neuromuscular or musculoskeletal disorders at the time of the study. The experimental procedures were approved by the National Medical Ethics Committee of the Republic of Slovenia and all the subjects gave informed consent before participation in the study.

**Figure 1.**
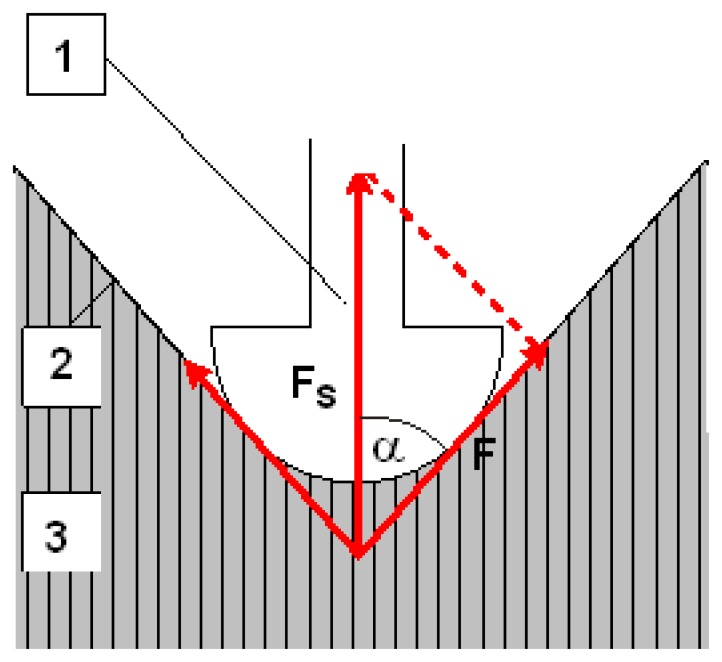
A simplified representation of the muscle contraction sensor (MC) measuring principle for the determination of the mechanical and physiological properties of skeletal muscles (1): Sensor tip; (2): Skin and intermediate layer; (3): Measured muscle.

The measurements were performed on the BB muscle under isometric conditions at two different elbow angles (15° and 90°) as shown in Figure 1. The subject sat in a custom-designed rig with the upper non-dominant arm next to the trunk, and the forearm in neutral horizontal position, semi-prone, with the palmar surface of the hand in the vertical plane. The angle of the elbow joint remained constant throughout the experimental session and was monitored with a high-speed camera (120 frames/s), placed perpendicular (side view) to the subject. The subject's shoulder was securely fastened to the back of the chair to prevent shoulder movement. A load cell (Mark 10, Portland, OR, USA) was fitted to the rig below the subject's wrist. A strap, attached to the load cell, was fastened around the subject's wrist.

The EMG, MC and force signals were measured simultaneously. We assumed that the measurements did not interfere with one another as the MC sensor was electrically isolated from the skin and could not have any significant effect on the electrical EMG signal. The muscle contraction being isometric meant that motion artefact was also unlikely to affect EMG readings.

### 2.1. MC Sensor

**Figure 2.**
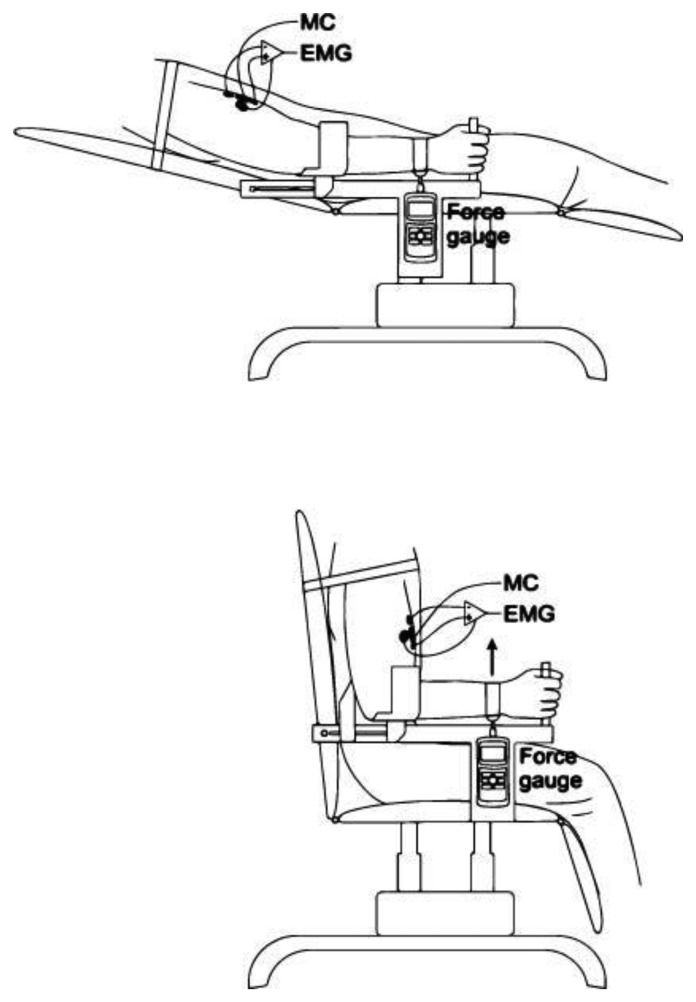
Measurement setup. Measurements were performed at two different elbow angles: 15° and 90°.

The MC sensor principle was presented in Sensors 2011 [27] and details can be seen in Figure 2. The supporting part of the MC sensor is made of elliptically shaped carbon fibre reinforced epoxy polymer. An incision forms a tonguelet to which the sensor tip is attached. The area of the part of the MC sensor attached to the skin is 650 mm^2^. Area of the sensor tip applying pressure to the skin is 56 mm^2^ and the length of the sensor tip indented in the skin fold is 5 mm. A piezo-resistive strain gauge is attached at the root of the tonguelet. The strain at the root of the tonguelet is proportional to the force acting on the sensor tip; the resistance of the piezo-resistor is proportional to that force. To compensate the temperature sensitivity of the semiconductor strain gauge, the resistance is measured with four piezo-resistors connected in a Wheatstone bridge.

The small size and lightweight design (<1 g) of the MC sensor enable continuous, unobtrusive monitoring of muscle tension. During measurement, the sensor is fixed on the skin above the muscle while the sensor tip shallowly indents the muscle. Muscle contraction produces tension in the longitudinal and perpendicular directions, the latter causing subcutaneous tissue and skin to press on the sensor tip.

### 2.2. EMG Recording

**Figure 3.**
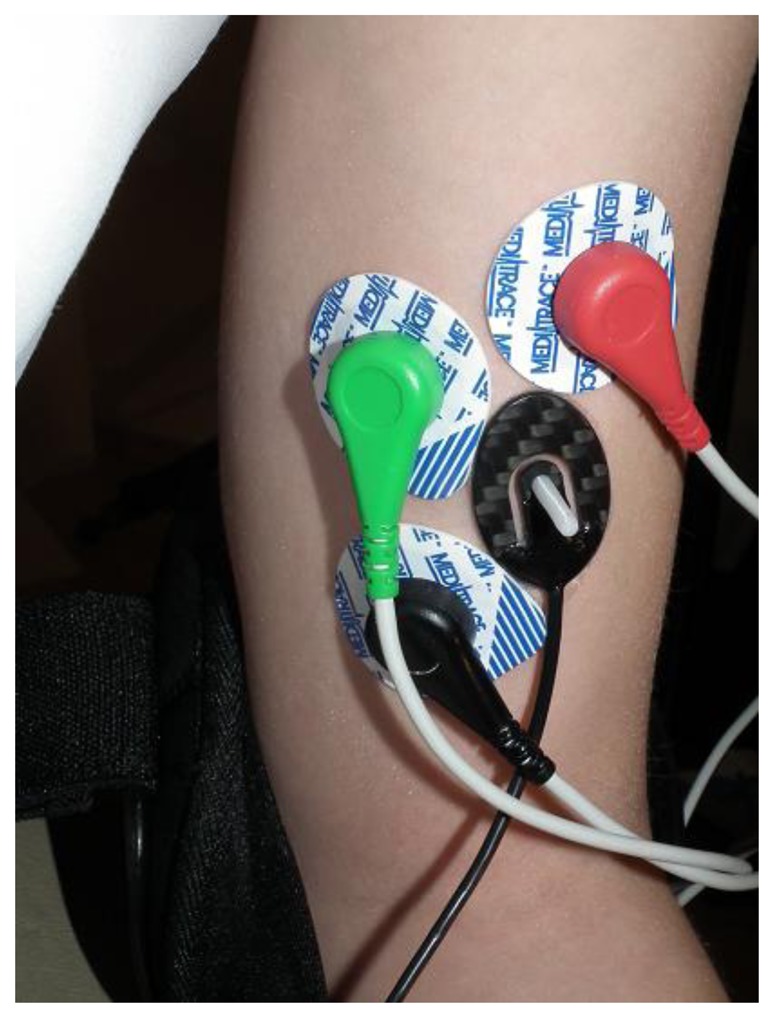
Position of MC sensor (black) and EMG electrodes on m. biceps brachii.

Before data collection, the skin over the proximal half of the muscle was prepared by gentle abrasion and cleansing with alcohol. A bipolar surface electrode arrangement was placed over the non-dominant *BB* muscle (Figure 3). The interelectrode distance was selected to accommodate the MC sensor. The recording electrodes were placed over the belly of the muscle, approximately midway between the auxiliary fold and the midpoint of the cubital fossa. The reference electrode was placed over the volar arch. The interelectrode impedance was kept below 5000 Ω through skin abrasion. The EMG signal was recorded using a 24-bit resolution, 25 m V/V NI 9237 module (National Instruments, Austin, TX, USA). We used MLA1010 Disposable ECG electrodes (AD Instruments, Australia).

### 2.3. MC Sensor Recording

The MC sensor was placed in the cross point of the longitudinal and transversal midlines of the left *BB* muscle (Figure 3). The signal from the MC sensor was again recorded with the NI 9237 module. The length of the sensor tip was the same for all subjects, irrespective of the tissue under the skin, which differed from subject to subject.

### 2.4. Force Recording

For force measurements, we used the digital force gauge Series 5 (Mark 10, Portland, OR, USA) with a sampling rate of 7 kHz and accuracy of ±0.1% of full scale. Analog output of the device was connected to the NI 9237 module.

### 2.5. Experimental Protocol

Each experimental session began with the subject performing trials of muscle voluntary contractions (MVCs) with the elbow flexor muscles at 15° and 90° elbow angles and with a 10 min rest period between contractions at both angles. The maximal force exerted by the subject during these two positions was used as the reference MVC force for the remainder of the experimental session.

The subjects could observe their performance on a monitor with a digital counter for each voluntary contraction trial.

For the measurement, the subjects were asked to perform three series of contractions continuously increasing from zero to 80% of the maximal force recorded in the trail, approximately 3 s in duration followed by releases, approximately 1 sec in duration. The timing relied on the verbal count given by the experimenter. A 10 min rest period was made between voluntary contractions at each elbow angle.

### 2.6. Signal Processing

Surface EMG (sEMG) served as the reference for muscle contraction onset. The sEMG signal was sampled at a 10 kHz sampling rate. Conditioning was adopted after Solnik *et al.* [28] and consisted of band-pass filtering at 30–300 Hz (6th order Butterworth filter) to remove motion artefacts, TKEO (Taeger-Kaiser energy operator), rectification and low-pass filtering at 50 Hz (2nd order Butterworth filter). Threshold was determined as a sum of mean and standard deviation of a 100 ms part of baseline. The estimated onset time was identified as the first point when the smoothed signal exceeded the threshold for more than 25 consecutive samples. After the band-pass noise filtering (20–450 Hz) [29] of the raw sEMG signal, the linear envelope (average rectified sEMG signal, 10 ms time interval, was smoothed using a 6th order low-pass Butterworth filter with cutoff frequency 10 Hz) seEMG procedure recommended by Merletti [30] was also calculated as an estimation of muscle force.

The MC signal was sampled at 10 kHz, filtered with 450 Hz cutoff frequency low-pass Gaussian filter, resampled at 1 kHz and calibrated [26] to show values in N. The force gauge signal was filtered with 450 Hz cutoff frequency low-pass Gaussian filter and resampled at 1 kHz.

### 2.7. Specific Signal Processing Related Statistics

In the paper from Đorđević *et al.* [27], it was shown that relationship between forces *F_MC_* measured with MC sensor and *F_D_* measured with dynamometer is nearly linear in static conditions, so that it holds:

(1) $$F_{MC}\approx kF_{D}$$

where *k* is the sensitivity of the system.

In this paper, we would like to explore whether this nearly linear relationship also holds when forces dynamically change during voluntary isometric contraction, *i.e.*, that:

(2) $${\text{F}}_{\text{MC}}\approx k{\text{F}}_{\text{D}}$$

where **F**_MC_ and **F**_D_ signal vectors of forces measured with MC and dynamometer, respectively. In order to show that, we calculate cross-correlation *R* of **F**_MC_ and **F**_D_. If *R* is high, we conclude that the relationship is nearly linear.

If we want to determine **F**_MC_ from the measurement of **F**_D_ using Equation (2) we must first find sensitivity *k* which minimizes mean square error:

(3) $$MSE(k)=\frac{\left({\text{F}}_{\text{MC}}-k{\text{F}}_{\text{D}})\cdot ({\text{F}}_{\text{MC}}-k{\text{F}}_{\text{D}}\right)}{N}$$

where *N* denotes the length of signal vectors **F**_MC_ and **F**_D_. *MSE*(*k*) is minimal when its first derivative is equal to zero:

(4) $$\frac{\partial MSE(k)}{\partial k}=\frac{2(k{\text{F}}_{\text{D}}-{\text{F}}_{\text{MC}})\cdot {\text{F}}_{\text{D}}}{N}=0$$

and

(5) $$k_{opt}=\frac{{\text{F}}_{\text{MC}}\cdot {\text{F}}_{\text{D}}}{{\text{F}}_{\text{D}}\cdot {\text{F}}_{\text{D}}}$$

Substituting *k_opt_* from Equation (5) for *k* in Equation (3) yields minimal MSE. Standard deviation of error σ can now be expressed as:

(6) $$\sigma =\sqrt{MSE(k_{opt})}$$

As a measure of linearity we can use with maximal value **F**_MC_ normalized standard deviation:

(7) $$\sigma_{N}=\frac{\sigma }{\max({\text{F}}_{\text{MC}})}$$

Small σ*_N_*, when forces dynamically change in time, also indicates good dynamical behaviour of the MC sensor itself, as it is capable to dynamically follow changing muscle contraction.

MATHEMATICA (Wolfram Research, Champaign IL, USA*)* and MATLAB (MathWorks, Natick MA, USA) were used for calculation and statistical analysis.

## 3. Results and Discussion

### 3.1. MC-F Signal Relationship

**Figure 4.**
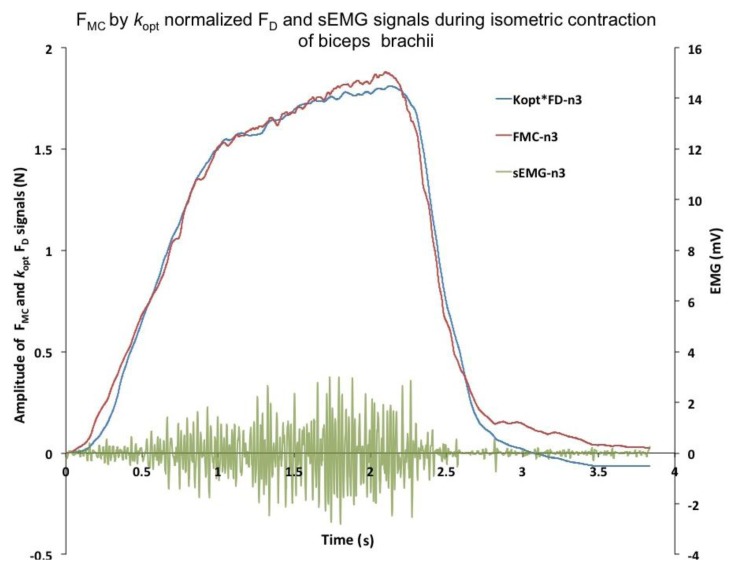
Simultaneously recorded *F_MC_*, *F_D_* and *EMG* during isometric contraction of biceps brachii muscle of subject 3 (n3). Coefficient sensitivity of the system *k*_opt_ (see Section 2.7) was used for normalization of *F*_D_ signal (*F*_MC_ ≈ *kF*_D_).

**Figure 5.**
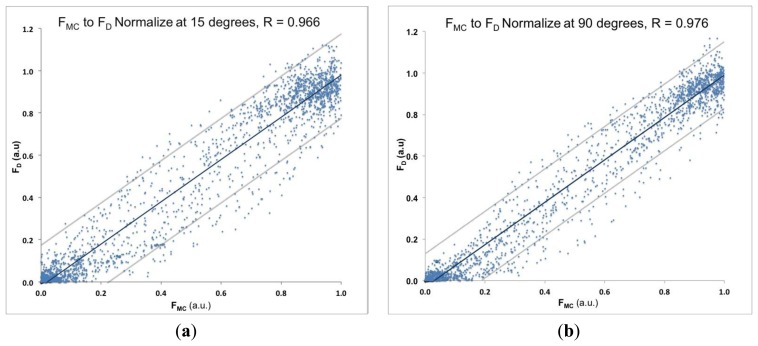
(**a**) Scatter plot of normalized *F_MC_* an *F_D_* signal at 15° elbow flexion with regression line and confidence bands at 0.95. Correlation coefficient was *R* = 0.966; (**b**) Scatter plot of normalized *F_MC_* an *F_D_* signal at 90° elbow flexion with regression line and confidence bands at 0.95. Correlation coefficient was *R* = 0.976.

The MC measurements were performed on the BB muscle, which is the strongest elbow flexor, while the dynamometer (*F_D_*) measurements were performed simultaneously on the wrist (Figure 1). The typical recording of *F_D_*, *F_MC_* and *EMG* is presented in Figure 4. The elbow angle was fixed at 90° and 15° angles. Typical recorded signal of *F_D_*, *F_MC_* and *EMG* measurement of isometric contraction of BB muscle during elbow flexion of 90° is shown on Figure 1. The strength of the linear relationship between *F_MC_* and *F_D_* was evaluated with a Pearson product–moment correlation coefficient *R* for each measurement and an *R*^2^ coefficient of determination. Very high *R* values *R* = 0.976 and *R* = 0.966 at 90° and 15°, respectively, indicate a strong linear relationship between these measured variables in both measured positions of the elbow (see Figure 5a correlation for 15° and Figure 5b for 90°).

Furthermore, the strength of the linear relationship for all *F_MC_* and *F_D_* data was defined by calculating the coefficient of determination *R^2^* which was 0.94 for elbow angle 90° and 0.93 for an elbow angle of 15°. This may be interpreted as approximately 93% of the variation for an elbow angle of 15° in the response variable *F_D_* which can be explained by the explanatory variable *F_MC_* and 94% of the variation between the response and the explanatory variable for elbow angle 90° can be explained by the linear relationship model. We also found a similar highly statistically significant linear relationship 0.99 ≥ *R*^2^ ≥ 0.97 between MC raw signal and force signal in the intermediate angles (15, 30, 45, 60, 75, and 90 degrees, see example in Supplementary Data, Figures S1–7).

For the relation *F*_MC_ ≈ *kF*_D_ the sensitivity of the system *k_opt_* was calculated for each elbow angle by method-specific signal processing related statistics. Average *k_opt_* for elbow angle 90° was *k_90_* = 0.00598 ± 0.00658 (average ± SD) and, for 15°, *k_15_* = 0.00918 ± 0.00309. *k_15_* and *k_90_* are statistically different (t-test) in spite of high variability (SD). This can be attributed to the diversity of conditions (muscle length, *k_15_* and *k_90_*) and histo-anatomical/morphological differences between subjects (high relative SD).

Normalized SD σ*_N_* used as a measure of linearity in dynamic conditions in Equation (7) was for all measurements below 20%, with average 8.24% for elbow angle 90° and 10.01% for an elbow angle of 15°, which indicates high linearity and good dynamic properties of MC sensor.

We compared also *seEMG* and *F_D_*. Average σ*_N_* was 14.62% at elbow angle 15° and 13.28% at an elbow angle of 90°.

To evaluate possible influence on correlation/connection between *F_MC_* an *F_D_* signal, we tested different variables: Skin-fold, length between the fulcrum and the strain gauge fixation point, peak of isometric *F_D_*, peak of *F_MC_*. Statistically significant correlation was found between σ*_N_* and skin fold (*R* = 0.74).

**Figure 6.**
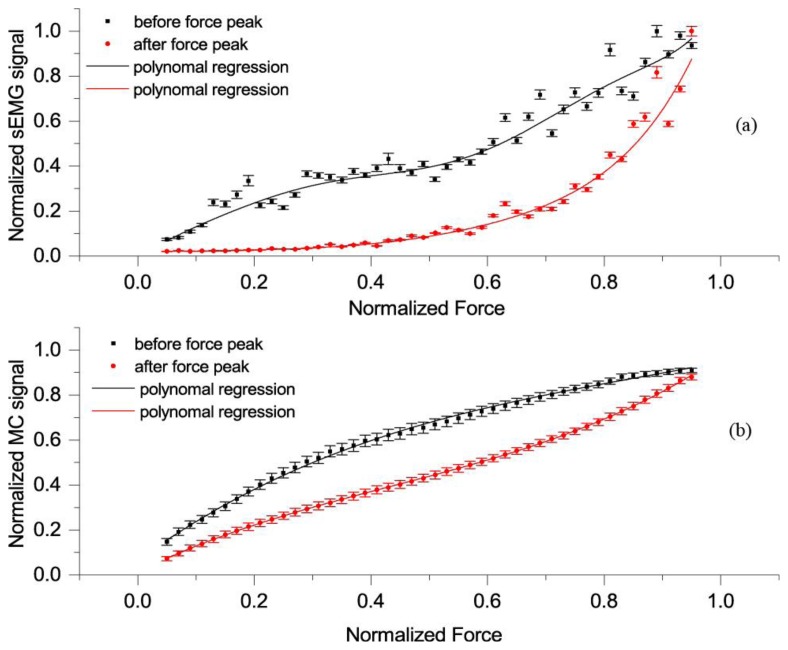
(**a**) Each point on curves is the average of all records of the mean normalized sEMG, obtained when *F_D_* was within 2% of each chosen force value (0.02, 0.4…to 0.98 at elbow angle 15°) while the force was rising (*i.e.*, before the peak, black colour) or while force was falling (*i.e.*, after the peak, red colour). Bars show SEM, and line is polynomial regression of the relationship between *F_MC_* and *F_D_*, before the peak (adjusted *R*^2^ = 0.96) and red line after the peak (adjusted *R*^2^ = 0.96); (**b**) Each point on the curves is the average of all records of the mean normalized *F_MC_*, obtained when *F_D_* was within 2% of each chosen force value (0.02, 0.4…to 0.98 at elbow angle 15°) while the force was rising (*i.e.*, before the peak, black colour) or while force was falling (*i.e.*, after the peak, red colour). Bars show SEM, and line is polynomial regression of the relationship between *F_MC_* and *F_D_*, before the peak (adjusted *R*^2^ = 0.99) and red line after the peak (adjusted *R*^2^ = 1).

Relation between force and MC signal before and after peak force was also analysed. In Figure 6a, we can see a relationship between normalized MC signal and force in rising force phase (*i.e.*, before the peak) and while force was falling (*i.e.*, after the peak). We can recognize a hysteresis-like relationship between normalized force and MC signal. The hysteresis is more pronounced at 15° than at 90° elbow angle.

The *F_D_*-sEMG diagram show an exponential relation and similar hysteresis as *F_D_*-*F_MC_*.

### 3.2. Discussion

The basic function of skeletal muscle is force production. Selective measuring and monitoring of the action of individual muscles is essential for understanding the function of muscle-tendon complex. It is very difficult to study the mechanical properties of individual skeletal muscles during normal action [31]. Monitoring muscle during movement is further complicated by the fact that significant muscle force is generated by active muscle contractions or due to passive muscle length changes.

Non-invasive and selective monitoring of mechanical properties of an individual muscle-tendon complex can help us study human motion and adaptation processes for different movement patterns/tasks. The first step in this direction is non-invasive measurement of muscle force/tension production in well-defined isometric conditions.

Previously, we showed a strong linear correlation between individual peak force measured by dynamometer and tension measured by MC sensor on BB muscle [27].

The aim of this study was to determine the dynamic properties of MC sensor in complete time domain of isometric contraction. We compared MC, force and seEMG signal during voluntary isometric contraction of BB at two elbow angles, 90° and 15°.

In short (90° elbow angle) and long muscle (15° elbow angle), we found statistically significant linear correlation between tension measured by MC and force measured by strain gauge, *R* = 0.971 and *R* = 0.961 at 90° and 15°, respectively. Linearity or correlation is similar in long and short muscle conditions.

Additionally, we tested linearity (MC-F) in time domain with normalized SD σ*_N_* as a measure of linearity in dynamic conditions in Equation (7). For all measurements, normalized SD had an average of 8.24% for elbow angle 90° and 10.01% for elbow angle 15°, which indicates a high linearity and good dynamic properties of the MC sensor.

Different sensitivity of the system *k_opt_* from subject to subject can be attributed to the morphological differences, length of the upper arm and forearm, different joint structure, skin fold, and other mechanical properties. Statistically different average *k_15_* and *k*_90_ (elbow joint 15° and 90°) may be due to different tensions/lengths of the muscle and the transforming function of the elbow joint. Transforming function is directly influenced by joint properties, which includes contributions from all structures located within and over the joint (muscles, tendons, skin, subcutaneous tissue, fascia, ligaments, joint capsule, and cartilage) [32–34].

*F_D_* signal was also compared to simultaneously measured *seEMG* signal. Average σ*_N_* was 14.62% at an elbow angle of 15° and 13.28% at an elbow angle of 90°. Both values for *seEMG-F_D_* relation are significantly higher in comparison with the *F_MC_*-*F_D_* relation (8.24% and 10.01%, respectively) meaning that the *F_MC_*-*F_D_* relation is a more linear fit than the *seEMG-F_D_* for the described measuring conditions. It is important to emphasize that EMG and MC are selective *in situ* measurements while dynamometry is not. Surface EMG and MC can be simultaneously utilized and give two different aspects of skeletal muscle action, electrical and mechanical.

It should also be noted that muscle force at a given level of muscle activity is affected by a range of factors not reflected in the *seEMG* signal, such as instantaneous muscle length and rate of length change [35] (Hof, 1997), contraction history [36] and fatigue [21,37].

**Figure 7.**
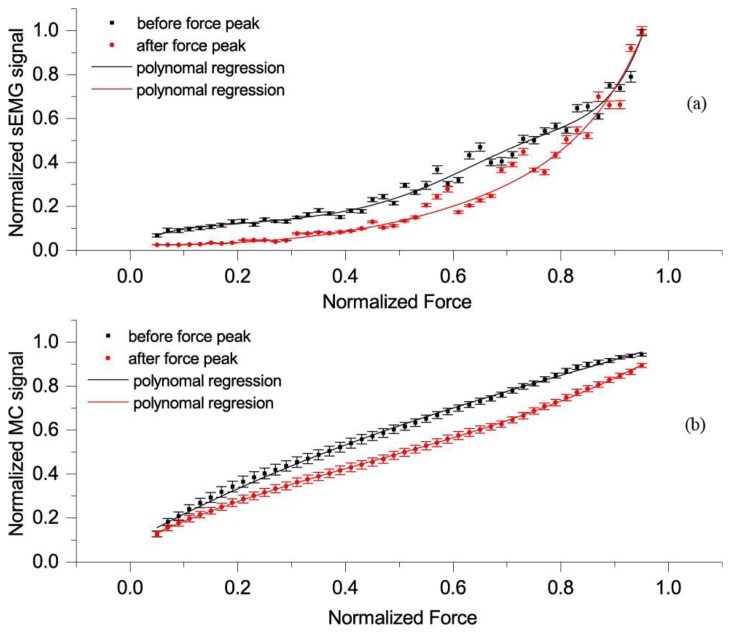
(**a**) Each point on curves is the average of all records of the mean normalized sEMG, obtained when *F_D_* was within 2% of each chosen force value (0.02, 0.4…to 0.98 at elbow angle 90°) while the force was rising (*i.e.*, before the peak, black colour) or while force was falling (*i.e.*, after the peak, red colour). Bars show SEM, and line is polynomial regression of the relationship between *F_MC_* and *F_D_*, before the peak (adjusted *R*^2^ = 0.96) and red line after the peak (adjusted *R*^2^ = 0.96); (**b**) Each point on curves is the average of all records of the mean normalized *F_MC_*, obtained when *F_D_* was within 2% of each chosen force value (0.02, 0.4…to 0.98 at elbow angle 90°) while the force was rising (*i.e.*, before the peak, black colour) or while force was falling (*i.e.*, after the peak, red colour). Bars show SEM, and line is polynomial regression of the relationship between *F_MC_* and *F_D_*, before the peak (adjusted *R*^2^ = 0.99) and red line after the peak (adjusted *R*^2^ = 0.99).

We also identified a hysteresis in the *F_D_-F_MC_* mean curve (Figure 6a,b), similar to *F_D_-seEMG* (Figure 7a,b), which was more pronounced at a 15° than at a 90° elbow angle. This hysteresis could be attributed to the friction of the elbow joint, sliding elements, antagonistic muscle stretch, tendon conditioning induced by contraction [38] and/or mechanical losses in muscle-tendon complex [39,40]. In the ascending phase, all these resist the muscle force, while in the descending phase these help the muscle to resist the force of dynamometer.

Also, a different approach can be applied to understand the importance of radial direction deformation (of which the MC sensor measures) of muscle during contraction. In a recent study [9,10], it was shown that lattice spacing inside actin myosin three-dimensional complex is a significant force regulator, increasing the slope of muscle's force–length dependence and the storage of elastic energy. The slope and shape of the length tension curve is a product of both the axial and the radial geometry of contracting muscles.

The sensitivity factor *k_opt_*, which we introduced to calculate standard deviation, is different from subject to subject and may also change with time. It depends on different properties of the subject and may also be used to establish some of them, which is a matter of further research.

### 3.3. Limitations of the Study

Unfortunately, there is no direct method for non-invasive measurement of muscle tension to compare to, thus we decided to compare MC readings to the force produced by the muscle of interest. However, this force depends on many different factors: morphological differences, length of upper and forearm, different joint structure, and contribution of other muscles.

On the other hand, the readings of MC sensor depend also on skin fold, subcutaneous thickness, and other mechanical properties.

From this, we can conclude that MC sensor cannot be used for measurement of the absolute value of muscle contraction. However, the large linear correlation between the MC and force signal shows that MC sensor dynamically follows the contractions and is thus suitable for dynamic measurement during voluntary free motion, when force cannot be measured.

## 4. Conclusions

MC signal indicated high linear correlation in the time domain with force measured during elbow isometric flexion. It seems that MC can be used to measure dynamic tension changes in a single muscle-tendon complex. There is consistency in the hysteresis-like relationship between force-MC and force-*seEMG*. Force-MC dependency was significantly more linear then force-*seEMG*.

A combination of selective mechanical and electrical information about skeletal muscle behaviour can give us additional understanding of mechanical function, adaptation properties and task dependency of the most common human body tissues.

Due to their small size and weight and their non-invasive properties, MC sensors can be applied in applications where wearable sensors are needed. One or many of them could be integrated in special cloths or multiple, separate, double-sided adhesive patches (for more selective recording of the muscle parts, or less unobtrusive and miniaturized solution during fast movements) for different purposes. One of the major applications can be monitoring the muscle-tendon complex behaviour during different movement patterns in normal or pathological conditions.

### Further Work

Future development and application of MC sensor/technology should go in two directions:

- Testing the application of MC sensor for tendon force/tension measurement (preliminary study [41])
- Testing the MC sensor in voluntary movement in different tasks, with different load intensity and velocity of muscle contraction.

Frequently, unobtrusive measurement of patients, healthy subjects or sportsmen can open up completely new approaches to monitoring, diagnostics of muscle tendon complex adaptations, and pathologies related to everyday activities, aging and rehabilitation/medical treatment. When fully tested, MC sensors will have such potential, and open up many new research topics.

## Supplementary Material



## References

[1] Lieber R.L. (2010). Methods for Human Muscle Force Determination, Skeletal Muscle Structure, Function, Plasticity.

[2] Schantz P., Randall-Fox E., Hutchison W., Tyden A., Astrand P.O. (1983). Muscle fibre type distribution, muscle cross-sectional area and maximal voluntary strength in humans. Acta Physiol. Scand..

[3] Rugg S.G., Gregor R.J., Mandelbaum B.R., Chiu L. (1990). *In vivo* moment arm calculations at the ankle using magnetic resonance imaging (MRI). J. Biomech..

[4] Ito M., Akima H., Fukunaga T. (2000). *In vivo* moment arm determination using b-mode ultrasonography. J. Biomech..

[5] Magnusson S.P., Aagaard P., Dyhre-Poulsen P., Kjaer M. (2001). Load-Displacement properties of the human triceps surae aponeurosis *in vivo*. J. Physiol..

[6] Gregor R.J., Roy R.R., Whiting W.C., Lovely R.G., Hodgson J.A., Edgerton V.R. (1988). Mechanical output of the cat soleus during treadmill locomotion: *In vivo vs. in situ* characteristics. J. Biomech..

[7] Komi P.V., Salonen M., Järvinen M., Kokko O. (1987). In vivo registration of Achilles tendon forces in man. I. Methodological development. Int. J. Sports Med..

[8] Komi P.V., Belli A., Huttunen V., Bonnefoy R., Geyssant A., Lacour J.R. (1996). Optic fibre as a transducer of tendomuscular forces. Eur. J. Appl. Physiol. Occup. Physiol..

[9] Williams C., Salcedo M., Irving T., Regnier M., Daniel T. (2013). The length-tension curve in muscle depends on lattice spacing. Proc. Biol. Sci..

[10] George N.T., Irving T.C., Williams C.D., Daniel T.L. (2013). The cross-bridge spring: Can cool muscles store elastic energy?. Science.

[11] Aagaard P., Simonsen E.B., Andersen J.L., Magnusson P., Dyhre-Poulsen P. (2002). Increased rate of force development and neural drive of human skeletal muscle following resistance training. J. Appl. Physiol..

[12] Baker D., Wilson G., Carlyon B. (1994). Generality *versus* specificity: A comparison of dynamic and isometric measures of strength and speed-strength. Eur. J. Appl. Physiol. Occup. Physiol..

[13] Hakkinen K., Komi P.V., Alen M. (1985). Effect of explosive type strength training on isometric force- and relaxation-time, electromyographic and muscle fibre characteristics of leg extensor muscles. Acta Physiol. Scand..

[14] Thorstensson A., Karlsson J., Viitasalo J.H., Luhtanen P., Komi P.V. (1976). Effect of strength training on emg of human skeletal muscle. Acta Physiol. Scand..

[15] Viitasalo J.T., Komi P.V. (1978). Force-Time characteristics and fiber composition in human leg extensor muscles. Eur. J. Appl. Physiol. Occup. Physiol..

[16] Hakkinen K., Alen M., Komi P.V. (1985). Changes in isometric force- and relaxation-time, electromyographic and muscle fibre characteristics of human skeletal muscle during strength training and detraining. Acta Physiol. Scand..

[17] Harridge S.D., Bottinelli R., Canepari M., Pellegrino M.A., Reggiani C., Esbjornsson M., Saltin B. (1996). Whole-Muscle and single-fibre contractile properties and myosin heavy chain isoforms in humans. Pflugers Arch. Eur. J. Physiol..

[18] Aagaard P. (2003). Training-Induced changes in neural function. Exerc. Sport Sci. Rev..

[19] Van Cutsem M., Duchateau J., Hainaut K. (1998). Changes in single motor unit behaviour contribute to the increase in contraction speed after dynamic training in humans. J. Physiol..

[20] Wilkin D.J. (1950). The relation between force and velocity in human muscle. J. Physiol..

[21] Lieber R.L., Leonard M.E., Brown-Maupin C.G. (2000). Effects of muscle contraction on the load-strain properties of frog aponeurosis and tendon. Cells Tissues Organs.

[22] Maganaris C.N., Narici M.V., Reeves N.D. (2004). *In vivo* human tendon mechanical properties: Effect of resistance training in old age. J. Musculoskelet. Neuronal Interact..

[23] Magnusson S.P., Hansen P., Aagaard P., Brond J., Dyhre-Poulsen P., Bojsen-Moller J., Kjaer M. (2003). Differential strain patterns of the human gastrocnemius aponeurosis and free tendon, *in vivo*. Acta Physiol. Scand..

[24] Reeves N.D., Maganaris C.N., Narici M.V. (2003). Effect of strength training on human patella tendon mechanical properties of older individuals. J. Physiol..

[25] Staudenmann D., Roeleveld K., Stegeman D.F., van Dieen J.H. (2010). Methodological aspects of semg recordings for force estimation—A tutorial and review. J. Electromyogr. Kinesiol. Off. J. Int. Soc. Electrophysiol. Kinesiol..

[26] Milner-Brown H.S., Stein R.B. (1975). The relation between the surface electromyogram and muscular force. J. Physiol..

[27] Dordevic S., Stancin S., Meglic A., Milutinovic V., Tomazic S. (2011). Mc sensor—A novel method for measurement of muscle tension. Sensors.

[28] Solnik S., DeVita P., Rider P., Long B., Hortobagyi T. (2008). Teager-Kaiser operator improves the accuracy of emg onset detection independent of signal-to-noise ratio. Acta Bioeng. Biomech. Wroclaw Univ. Technol..

[29] De Luca C.J., Gilmore L.D., Kuznetsov M., Roy S.H. (2010). Filtering the surface emg signal: Movement artifact and baseline noise contamination. J. Biomech..

[30] Merletti R., Parker P., Merletti R., Hermens J. (2004). Detection and Conditioning of the Surface EMG Signal. In Electromyography: Physiology, Engineering, and Noninvasive Applications.

[31] Davis J., Kaufman K.R., Lieber R.L. (2003). Correlation between active and passive isometric force and intramuscular pressure in the isolated rabbit tibialis anterior muscle. J. Biomech..

[32] Riemann B.L., DeMont R.G., Ryu K., Lephart S.M. (2001). The effects of sex, joint angle, and the gastrocnemius muscle on passive ankle joint complex stiffness. J. Athl. Train..

[33] Wright V. (1973). Stiffness: A review of its measurement and physiological importance. Physiotherapy.

[34] Helliwell P.S., Wright V., Radin E.L. (1993). Joint Stiffness. Mechanics of Joints: Physiology, Pathophysiology and Treatment.

[35] Hof A.L. (1997). The relationship between electromyogram and muscle force. Sportverletz. Sportschaden.

[36] Welter T.G., Bobbert M.F. (2001). During slow wrist movements, distance covered affects EMG at a given external force. Motor Control.

[37] Lind A.R., Petrofsky J.S. (1979). Amplitude of the surface electromyogram during fatiguing isometric contractions. Muscle Nerve.

[38] Maganaris C.N. (2003). Tendon conditioning: Artefact or property?. Proc. Biol. Sci..

[39] Stokes I.A. (2005). Relationships of EMG to effort in the trunk under isometric conditions: Force-Increasing and decreasing effects and temporal delays. Clin. Biomech..

[40] Solomonow M. (2009). Ligaments: A source of musculoskeletal disorders. J. Bodyw. Mov. Ther..

[41] Djordjević S., Meglič A., Morrissey D., Langberg H., Tomazič S., Meeusen R., Duchateau J., Roelands B., Klass M., de Geus B., Baudry S., Tsolakidis E. (2012). Noninvasive Measurements of the Tension Developed in the Patellar Tendon during Squatting—A Case Study with a Novel Sensor. Muscle-Tendon-Bone.

