# Supplementary material for: In-Vivo Measurement of Muscle Tension: Dynamic Properties of the MC Sensor during Isometric Muscle Contraction
Source: Sensors (Basel). 2014 Sep 25;14(9):17848–63. doi: 10.3390/s140917848 (PMC4208254; doi:10.3390/s140917848)
Supplement: Supplementary file 1 [file sensors-14-17848-s001.pdf]

OPEN ACCESS

sensors

ISSN 1424-8220

www.mdpi.com/journal/sensors

Supplementary Information

In-Vivo Measurement of Muscle Tension: Dynamic Properties of the MC Sensor during Isometric Muscle Contraction. *Sensors* 2014, *14*, 17848-17863

Srđan Đorđević [1,2,*], Sašo Tomažič [3], Marco Narici [4], Rado Pišot [5] and Andrej Meglič [1]

[1] TMG-BMC Ltd., Splitska 5, Ljubljana 1000, Slovenia; E-Mail: andrej.meglic.info@gmail.com

[2] Institute for Kinesiology Research, Science and Research Centre of the University of Primorska, Garibaldijeva 1, Koper 6000, Slovenia

[3] Faculty of Electrical Engineering, University of Ljubljana, Tržaška 25, Ljubljana 1000, Slovenia; E-Mail: saso.tomazic@fe.uni-lj.si

[4] University of Nottingham, School of Graduate Entry Medicine and Health, Derby Royal Hospital, Uttoxeter Road, Derby DE22 3DT, UK; E-Mail: marco.narici@nottingham.ac.uk

[5] Institute for Kinesiology Research, Science and Research Centre of the University of Primorska, Garibaldijeva 1, Koper 6000, Slovenia; E-Mail: rado.pisot@zrs.upr.si

* Author to whom correspondence should be addressed; E-Mail: srdjand@tmg.si; Tel.: +38-641-672-601; Fax: +38-613-007-777.

For a few subjects, we also tested the force to MC sensor relationship using the same protocol for intermediate angles 15°, 30°, 45°, 60°, 75° and 90°. Representative normalized raw data of one subject are presented in Figures S1–S6. The coefficient of determination R^2 was high and similar in all angles between 0.97 and 0.99. Figure S7 presents the slopes and range (related to raw data) of regression lines (k_S) at elbow angles 90°, 75°, 60°, 45°, 30°, 30°, 15°.

Figure S1. Normalized raw F and MC signal from three repetitions at elbow angle 90° from one subject.

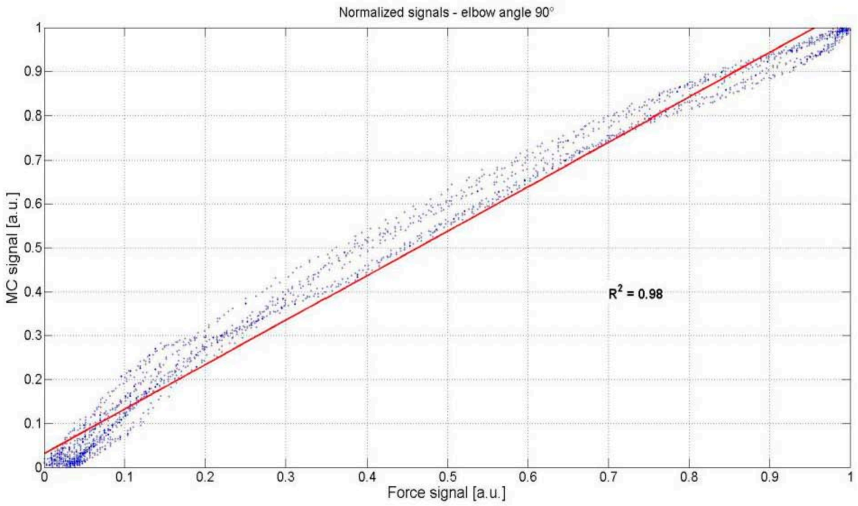

Figure S2. Normalized raw F and MC signal from three repetitions at elbow angle 75° from one subject.

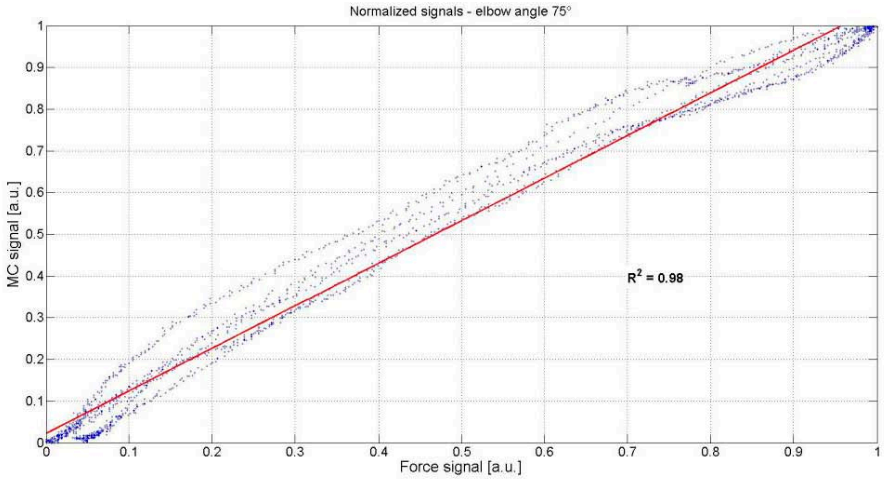

Figure S3. Normalized raw F and MC signal from three repetitions at elbow angle 60° from one subject.

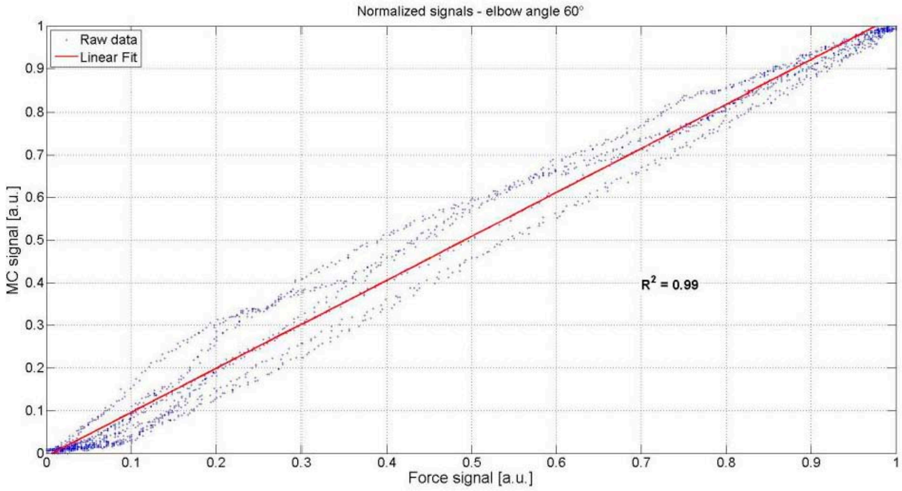

Figure S4. Normalized raw F and MC signal from three repetitions at elbow angle 45° from one subject.

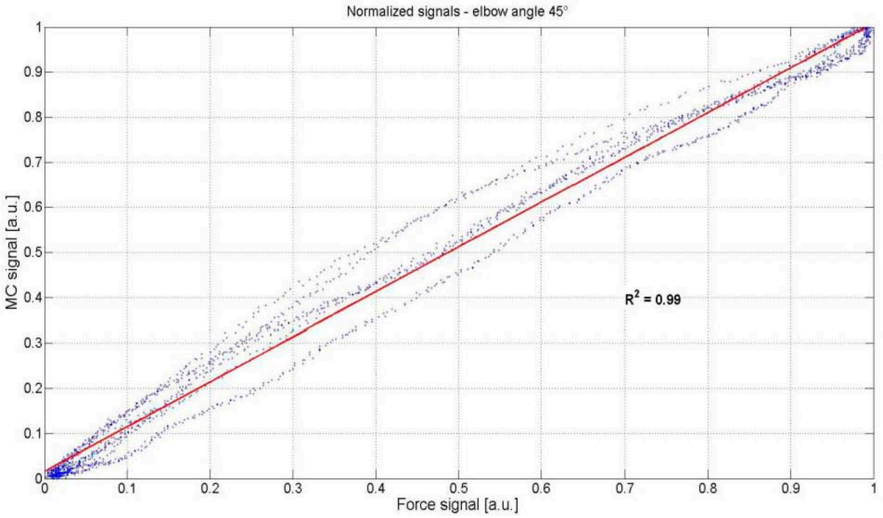

Figure S5. Normalized raw F and MC signal from three repetitions at elbow angle 30° from one subject.

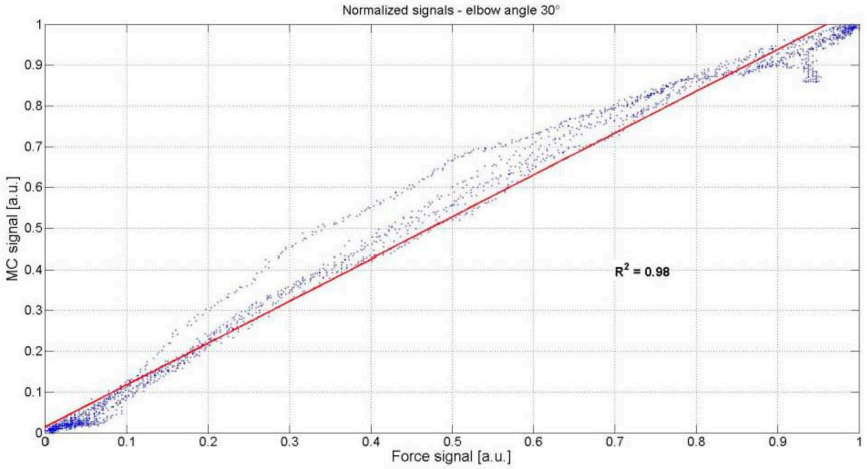

Figure S6. Normalized raw F and MC signal from three repetitions at elbow angle 15° from one subject.

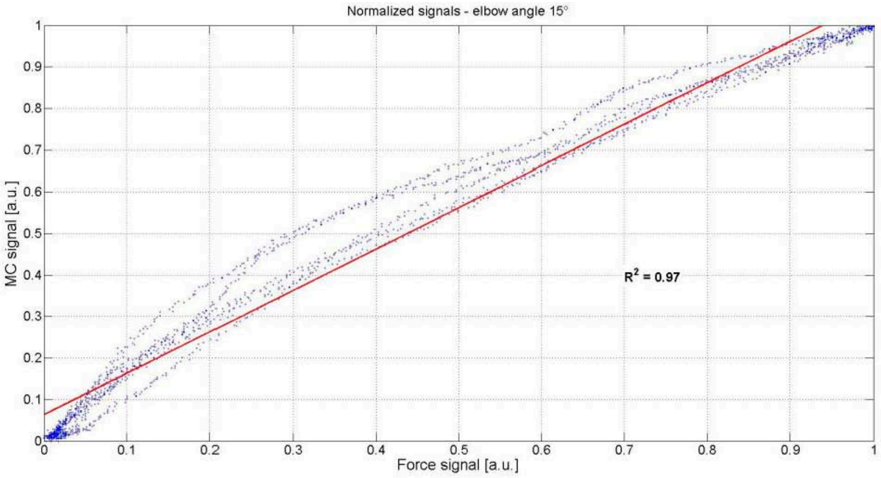

Figure S7. Regression lines were calculated from normalized raw F and MC data. MC15, MC30, MC45, MC60, MC75 and MC90 are regression lines (real range) for elbow angles 15°, 30°, 45°, 60°, 75° and 90°, respectively. k_s is the slope of the regression line. (a.u. = arbitrary units).

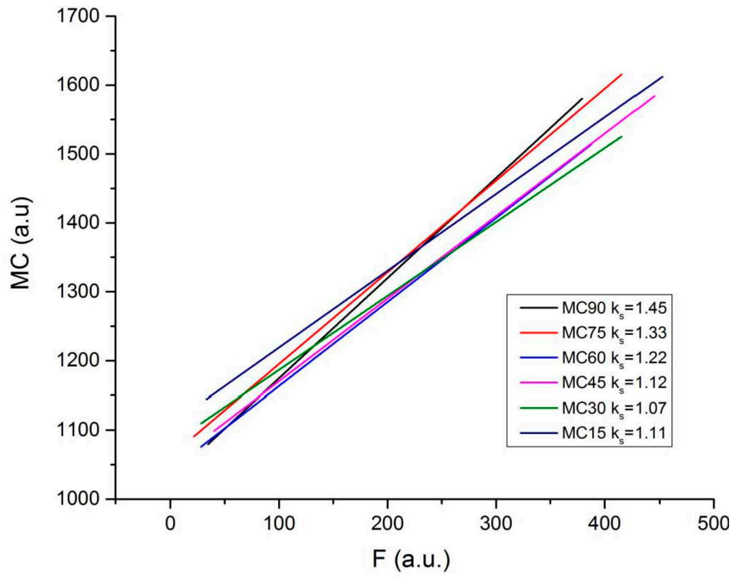

© 2014 by the authors; licensee MDPI, Basel, Switzerland. This article is an open access article distributed under the terms and conditions of the Creative Commons Attribution license (http://creativecommons.org/licenses/by/3.0/).
